# Role of altered proteostasis network in chronic hypobaric hypoxia induced skeletal muscle atrophy

**DOI:** 10.1371/journal.pone.0204283

**Published:** 2018-09-21

**Authors:** Akanksha Agrawal, Richa Rathor, Ravi Kumar, Geetha Suryakumar, Lilly Ganju

**Affiliations:** Defence Institute of Physiology and Allied Sciences, Timarpur, Delhi, India; University of Louisville School of Medicine, UNITED STATES

## Abstract

**Background:**

High altitude associated hypobaric hypoxia is one of the cellular and environmental perturbation that alters proteostasis network and push the healthy cell towards loss of muscle mass. The present study has elucidated the robust proteostasis network and signaling mechanism for skeletal muscle atrophy under chronic hypobaric hypoxia (CHH).

**Methods:**

Male Sprague Dawley rats were exposed to simulated hypoxia equivalent to a pressure of 282 torr for different durations (1, 3, 7 and 14 days). After CHH exposure, skeletal muscle tissue was excised from the hind limb of rats for biochemical analysis.

**Results:**

Chronic hypobaric hypoxia caused a substantial increase in protein oxidation and exhibited a greater activation of ER chaperones, glucose-regulated protein-78 (GRP-78) and protein disulphide isomerase (PDI) till 14d of CHH. Presence of oxidized proteins triggered the proteolytic systems, 20S proteasome and calpain pathway which were accompanied by a marked increase in [Ca^2+^]. Upregulated Akt pathway was observed upto 07d of CHH which was also linked with enhanced glycogen synthase kinase-3β (GSk-3β) expression, a negative regulator of Akt. Muscle-derived cytokines, tumor necrosis factor-α (TNF-α), interferon-ϒ (IFN-©) and interleukin-1β (IL-1β) levels significantly increased from 07d onwards. CHH exposure also upregulated the expression of nuclear factor kappa-B (NF-κB) and E3 ligase, muscle atrophy F-box-1 (Mafbx-1/Atrogin-1) and MuRF-1 (muscle ring finger-1) on 07d and 14d. Further, severe hypoxia also lead to increase expression of ER-associated degradation (ERAD) CHOP/ GADD153, Ub-proteasome and apoptosis pathway.

**Conclusions:**

The disrupted proteostasis network was tightly coupled to degradative pathways, altered anabolic signaling, inflammation, and apoptosis under chronic hypoxia. Severe and prolonged hypoxia exposure affected the protein homeostasis which overwhelms the muscular system and tends towards skeletal muscle atrophy.

## Introduction

Skeletal muscle is highly plastic and adaptable tissue, amenable to multitudinous stressors, cause serious changes in whole-body metabolism. Reduce oxygen availability at extreme altitude affects skeletal muscle and slackens physical performance. As one of the prime hassles at high altitude (HA) is hypobaric hypoxia, skeletal muscle redox homeostasis is disrupted with modified protein structure and function which then causes a change in the metabolic process, homeostasis, and contractile performance. Shreds of evidence from past studies suggested that an altitude of 5000m and higher (Everest Base camp) led to the considerable deterioration of skeletal muscle tissue [1]. Our recent study also provided the detailed overview of oxidative protein modification in response to hypobaric hypoxia, which altered muscle protein homeostasis and triggered ER stress [2]. Although the acute hypoxia exposure was activating several proteolytic pathways, studies have established that prolonged stay at high altitude leads to loss of skeletal muscle mass and a decline in physical performance [3, 4]. Sustained exposure to hypoxia is one of the major reason for the detrimental effects on skeletal muscle structure leading to skeletal muscle atrophy [5, 6].

Muscle mass is dynamically regulated by the precise equilibrium of protein synthesis and degradation. Recent studies highlighted a complex scenario whereby a convoluted network of signaling cascades regulates myofibre’s size and the contractile performance of muscle. Intriguingly, these different pathways crosstalk, modulate one another at different levels, coordinating protein synthesis and degradation simultaneously. Past study from our own lab have reported the fact that the protein degradation rate is much higher than the protein synthesis rate under CHH [6]. Muscle proteolysis is mediated via the coordination of several cellular networks that include oxidative stress [7], activation of calpains, and non-lysosomal proteases such as ubiquitin-proteasome system (UPS) [8]. On the other hand, insulin growth factor-1 (IGF)/Akt pathway maintains muscle mass and myofibril growth through nebulin and neural Wiskott-Aldrich syndrome protein (N-WASP) [9]. Few studies reported a decrease in protein synthesis in limb muscles including chronic obstructive pulmonary disease (COPD) patients, cancer cachexia and sarcopenia [10, 11]. However, few other studies presented contradictory results with no decrease in protein synthesis signaling [12].

Previously, few studies have reported about the pivotal role of inflammation during hypoxic conditions in a wide array of human diseases [13]. Available evidences illustrates that stimulation of TNF-α and IL-1β induce expression of the Mafbx-1 and MuRF-1,ubiquitin ligases via transcription factor nuclear factor-kappa B (NFκB) [14, 15]. However, the exact plausible mechanism remains elusive, how hypoxia elicited inflammation in skeletal muscles. Secondly, why and how these changes may progress at high altitude to more severe damage and muscle mass loss is still unclear.

Although recent studies have highlighted the role of different signaling pathways in skeletal muscle atrophy and no comprehensive mechanistic study on the chronic hypobaric hypoxia induced skeletal muscle loss is available till date. Although increase number of people are exposed to high altitude for prolonged period, however very few research reports are existing regarding the same. Hence, the present study was planned to establish the role of altered proteostasis in chronic hypobaric hypoxia-induced skeletal muscles atrophy.

## Materials and methods

### Ethics statement

All animal procedures and experimental protocols were reviewed and approved by the Institutional Animal Ethics Committee (IAEC) accredited to Committee for the Purpose of Control and Supervision of Experiments on Animals (CPCSEA), Government of India. All animal experiments were conducted in accordance with the National Institutes of Health (NIH) Guide for the Care and Use of Laboratory Animals.

Male Sprague-Dawley rats, weighing 220±10 g, bred in the animal facility of Defence Institute of Physiology and Allied Sciences (DIPAS), Delhi, were maintained on a bedding of rice husk in polypropylene cages under controlled environment in the Institute’s animal house at 25±1°C, 55±10% humidity, and 12-h light-dark cycle. Animals had access to standard rodent pellet feed and water ad libitum. All the experimental procedures were followed as per standard experimental procedures to minimize the suffering of animals.

The information regarding all the biochemical reagents and the antibodies used in the western blotting are mentioned in the supporting files (S1, S2 and S3 Tables).

### Hypobaric hypoxia exposure

Twenty five male Sprague Dawley rats were taken for the experimental purpose which was divided into five groups of five rats in each group:

Group 1 Untreated and unexposed to CHH rats served as control
Group 2 01 day hypobaric hypoxia exposure
Group 3 03 days hypobaric hypoxia exposure
Group 4 07 days hypobaric hypoxia exposure
Group 5 14 days hypobaric hypoxia exposure

Simulated high altitude exposure was performed in an animal decompression chamber maintained at pressure of 282 torr (equivalent to an altitude of 7620 m, 8% oxygen), coupled to mercury barometer, at 25°C for hypoxic group (Decibel Instruments, India). The airflow in the chamber was 2 l/min with relative humidity maintained at 45 to 55%. Control group rats were maintained in the normoxic condition within the same laboratory. On completion of CHH exposure, animals were anaesthetized with sodium pentobarbital (50 mg/kg, *i*.*p*.), rats were sacrificed and skeletal muscle from hind limb of rats was excised for biochemical and histopathological analysis. Muscle samples were snap frozen in liquid nitrogen and all samples were stored at −80°C.

### Wet skeletal muscle weight to the tibia length ratio

After completion of hypobaric hypoxia exposure, the rats were sacrificed and whole muscle from hind limb were isolated and weighed using a digital platform balance. The wet muscle weight was expressed as the wet skeletal muscle weight/tibia length.

### Muscle damage marker by ELISA

Creatine Phosphokinase (CPK) assay was measured in rat skeletal muscle homogenate using commercially available kit (Mybiosource, Inc) as per manufacturer’s instructions. CPK activity was expressed as ng/mg protein.

### Protein oxidation and modification marker

#### Advanced oxidation protein products (AOPPs)

AOPP considered as a relevant marker for oxidant induced protein damage. It is formed during oxidative stress by the action of chlorinated oxidants, mainly hypochlorous acid and chloramines. Determination of AOPP (i.e. some oxidation products with characteristic absorbance) was based on spectrophotometric detection modified for muscle tissue as previously described [16]. Concentration of AOPPs was expressed as μmol/ chloramine/ mg protein.

#### Protein carbonylation

Oxidative modifications of amino acid residues include derivatization of amino acid residues such as proline, arginine, and lysine to reactive carbonyl derivatives. Briefly, 2,4- Dinitrophenylhydrazine (DNPH) reacts with protein carbonyl forming a Schiff base to produce the corresponding hydrazone, which can be analyzed spectrophotometrically as previously described [17]. The concentration of carbonyl groups was calculated by using an absorbance coefficient 22 nM cm^-1^ and expressed as nmol carbonyl/ mg of protein.

### Protein degradation

#### 20S proteasome activity

The ubiquitin proteasome pathway (UPP) was studied by assaying the chymotrypsin-like enzyme activity of 20S Proteasome, as described earlier [18]. Fluorescence of the liberated AMC was monitored in a Perkin-Elmer fluorimeter at excitation 380 nm, emission 460 nm.

#### Calpain assay

Calpain activity was measured in the homogenate using N-succinyl-Leu-Tyr-7-amido-4-methylcoumarin (SLY-AMC) as a substrate, described earlier [19]. Fluorescence of the liberated AMC was monitored in a Perkin Elmer fluorimeter (LS45) at excitation 380 nm, emission 460 nm.

### Intracellular free calcium

Intracellular calcium was measured as described previously [20]. The intracellular free calcium was determined by fluorescent calcium indicator dye Fura-2/AM. Fura-2/AM crosses the membrane, and it is hydrolyzed by the esterases to Fura-2 that binds free ionic calcium to give fluorescence, which is proportional to the amount of free calcium. The fluorescence (F) at 340- to 380-nm excitation and 510-nm emission was measured, and [Ca^2+^]i was calculated as follows:

$${\left[{\mathrm{Ca}}^{2+}\right]}_{i}=\left[\left(F‑\mathrm{Fmin}\right)/\left(\mathrm{Fmax}‑F\right)\right]{\times K}_{d}$$

The K_d_ value for Ca^2+^ Fura-2 complex was 225 nm. Maximal fluorescence (Fmax) was measured after lysis of plasma membrane of skeletal muscles with SDS, and minimal fluorescence (Fmin) was measured in the presence of 5 mM EGTA. The results were expressed as nanomolar of free intracellular calcium.

### Insulin-like growth factor-1

IGF-1 was measured using commercially available rat ELISA kit (Diaclone, France) according to manufacturer’s instructions. The intensity of the color reaction was read spectrophotometrically in a plate reader and the concentration expressed in ng/mg protein.

### Pro- inflammatory cytokines

Pro-inflammatory cytokines were determined in muscle homogenate using commercially available ELISA kit. IL-1β, IFN-ϒ and TNF-α was done by respective ELISA kits (Diaclone, France) as per manufacturer’s instructions. The concentrations were expressed as pg/ mg protein.

### Apoptosis

#### Caspase-3 substrate cleavage assay

Caspase-3 is an enzyme activated during the induction of apoptosis. The activity of caspase 3 in skeletal muscle was estimated using colorimetric substrate, Ac-Asp-Glu-Val-Asp p-Nitroaniline, Ac-DEVD-pNA (Calbiochem) by previously described [21]. Results were calibrated with known concentrations of p-NA and expressed as nmol/ p-NA/ minute/mg protein.

#### Caspase-9 substrate cleavage assay

Caspase-9, cysteine proteases participates in activating the apoptotic cell death machinery. The activity of caspase 9 in skeletal muscle was estimated using colorimetric substrate II, Ac-Leu-Glu-His-Asp-pNA, Ac-DEVD-pNA (Calbiochem) by previously described [22]. Results were calibrated with known concentrations of p-NA and expressed as nmol/ p-NA/minute/ mg protein.

#### Annexin-V

Annexin-V was measured using commercially available rat annexin-V ELISA kit (Elabscience, CA, USA) according to manufacturer’s instructions. The intensity of the color reaction was read spectrophotometrically in a plate reader and the concentration expressed in ng/mg protein.

### Immunoblotting

#### Preparation of nuclear and cytoplasmic extracts

For cytoplasmic fraction, muscle tissue was homogenized in an ice-cold buffer (0.5 M sucrose, 10 mM HEPES, 10 mM KCl, 1.5 mM MgCl_2_, 10% glycerol, 1 mM EDTA, 1 mM DTT, 1 mM PMSF fortified with protease inhibitors). Homogenates were kept on ice for 15 min, 0.6% Nonidet P-40 added, and then centrifuged for 20 min at 5,000g at 4°C. The supernatant with cytoplasmic fraction was collected and stored, and the pellet was dissolved in ice cold buffer B (20mM HEPES, 1.5mM MgCl_2_, 0.3mM NaCl, 0.2mM EDTA, 20% glycerol, 0.5mM DTT, 0.5mM PMSF and cocktail of protease inhibitors) for the nuclear fraction. It was incubated for 30 min on ice followed by centrifugation at 20,000 g at 4°C for 15 min. The supernatant containing the nuclear fraction was aliquoted and stored at −80°C for further analysis. Total protein concentrations were determined using the Bradford method [23].

#### Western blotting

Protein (50 μg) was separated by 10% and 12% SDS-polyacrylamide gel electrophoresis, based on the molecular weight of the protein of interest and transferred onto a nitrocellulose membrane (Millipore, Billerica, USA). The membranes were blocked with 3% bovine serum albumin in PBS containing 0.1% Tween 20 (Sigma), washed and probed with respective mouse/rabbit monoclonal antibodies. Primary antibodies PDI, Akt, p-Akt and CHOP/GADD153 were obtained from Santa Cruz Biotech, p70S6kinase from Cell Signaling Technology (MA, USA), GRP-78, GSK-3β and NFκB from Sigma (St. Louis, MO, USA) while MAFbx-1 and MuRF-1 from abcam. The membranes were then incubated with anti-mouse/rabbit-IgG HRP conjugate (Sigma). The membrane was washed and incubated with chemiluminescent substrate (Sigma) and the bands were developed using Gel Documentation System (UVP Bioimaging software, Upland CA, USA). Quantification was performed by densitometry using ImageJ software. GAPDH from Sigma (St. Louis, MO, USA) was used as an internal (loading) control.

### Protein-protein networking by STRING 10.0

Protein-protein interactions network was done by STRING 10.0 Software (http://string.embl.de).

### Statistical analysis

All the experiments were performed on a minimum of three different occasions, and data are presented as mean±SEM. One-way analysis of variance with post hoc Bonferroni analysis was used to determine statistical significance between groups. All analysis was conducted using GraphPad Prism ver 7 software (GraphPad, CA, USA). The p value of ≤0.05, with a 95% confidence interval was considered significant.

## Result

### Chronic hypobaric hypoxia induced skeletal muscle damage and atrophy

**Fig 1:**
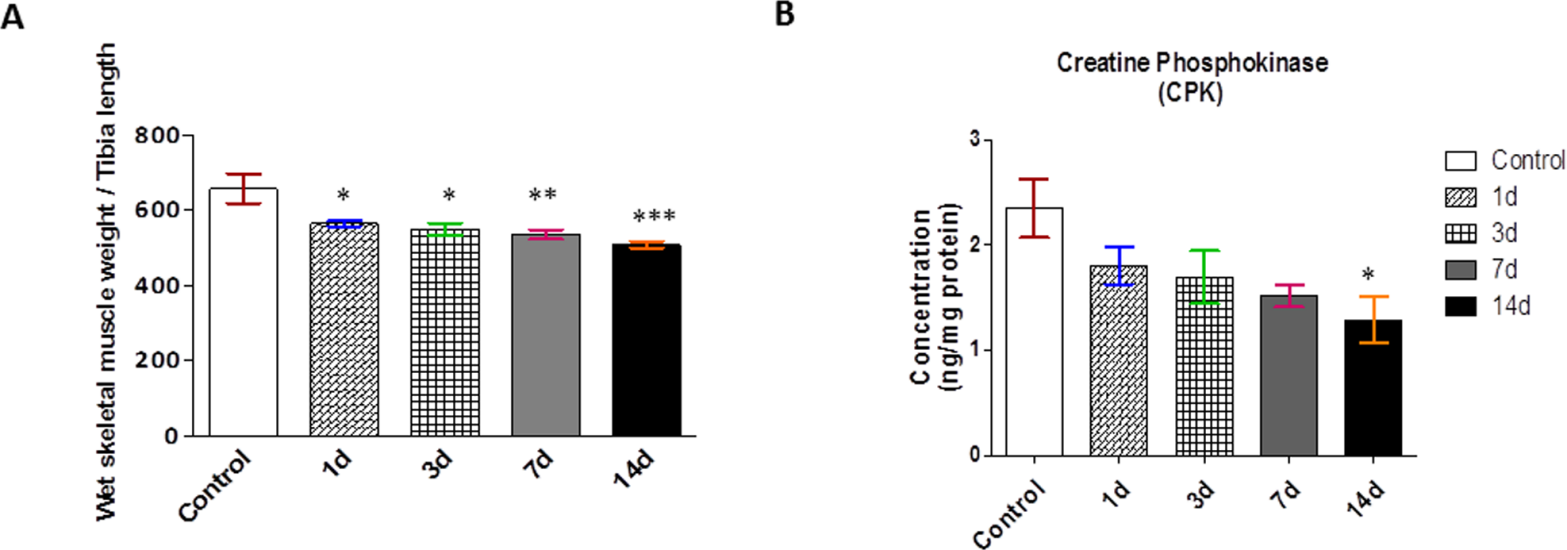
Effect of chronic hypoxia on appendicular body parameters. **(A)** Ratio of skeletal muscle weight & tibial length of rats **(B)** Creatine phosphokinase (CPK) *p<0.05 Vs. Control; **p<0.01 Vs. Control; ***p<0.001 Vs. Control.

CHH exposed rats, displayed significantly lower skeletal muscle weights and tibia length ratio as compared to control rats. A decrement was observed by 18.5% on 07days CHH exposure which was further moved up to 23% decrease till 14 days CHH exposure, indicating the time-dependent muscle mass loss. (Fig 1A). The severity of skeletal muscle damage in response to CHH was also measured in terms of CPK level in muscle. Disruption of the sarcolemma increased membrane permeability which allows the release of creatine kinase from the muscle’s cytoplasm into the bloodstream. Thus, high level of CPK in circulation and lower level of CPK in muscle tissue is an indicator of skeletal muscle damage. A significant decrease of 45% in the CPK content in muscle homogenate was noted in 14 days CHH exposed rats in relation to control rats (Fig 1B).

### Oxidized/misfolded proteins activate protein degradative pathways and ER chaperones

**Fig 2:**
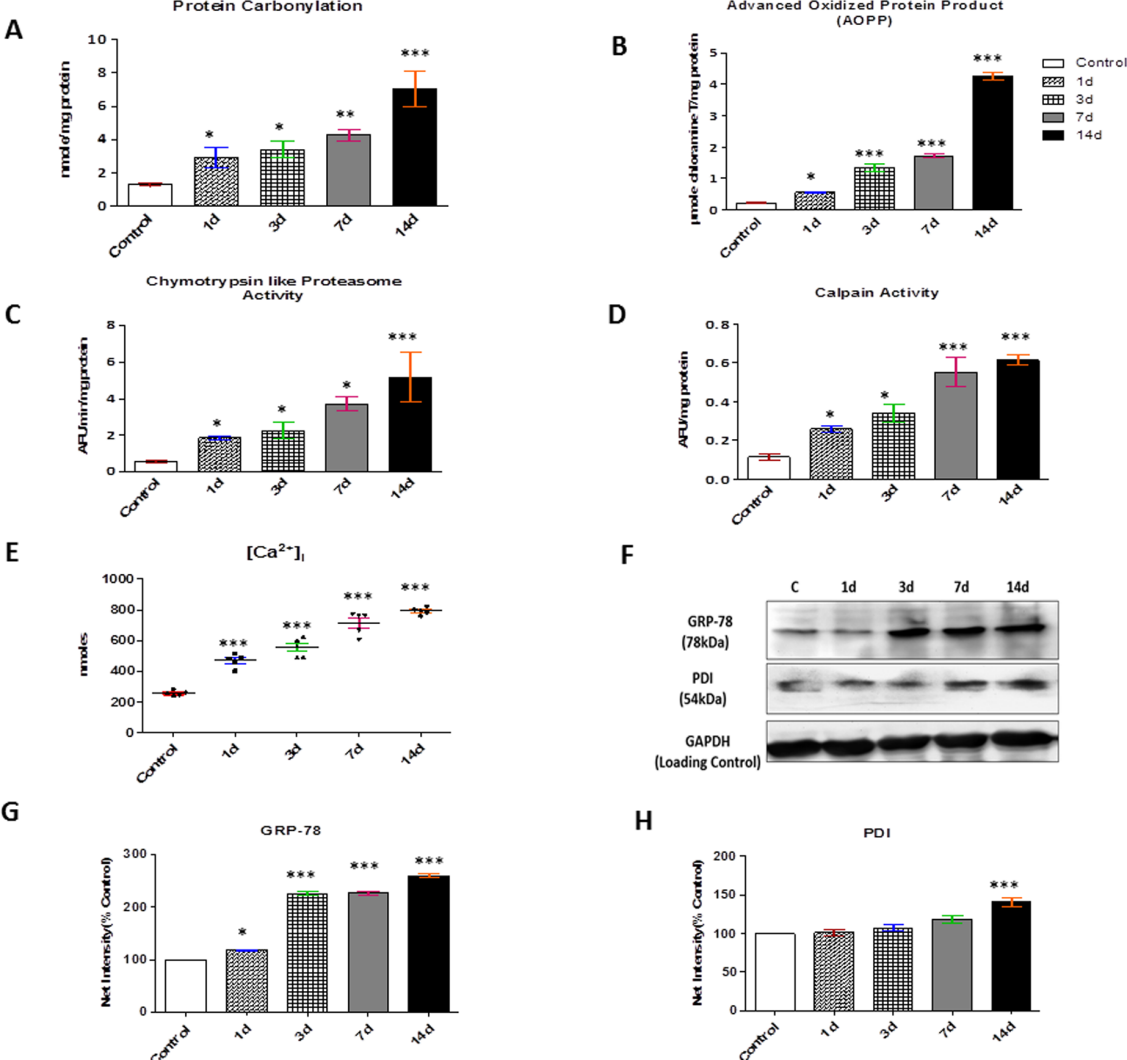
Presence of oxidized/misfolded proteins activates protein degradative pathways. **(A)** Protein Carbonylation **(B)** Advanced Oxidized protein product (AOPP) **(C)** Chymotrypsin-like 20 proteasome activity **(D)** Calpain activity **(E)** Intracellular Calcium ion **(F)** Representative western blots for expression of ER chaperones of skeletal muscle cytoplasmic extracts. (n = 3) and **(G-H)** Semiquantitative analysis of the expression of GRP-78 and PDI. GAPDH considered as the loading control. The densitometric analysis is shown as mean with standard error (bars) performed in n = 3 independent experiments. *p<0.05 Vs. Control; **p<0.01 Vs. Control; ***p<0.001 Vs. Control.

Multiple forms of ROS lead to the formation of peptide fragments possessing highly reactive carbonyl groups (ketones, aldehydes) and presence of protein carbonyl derivatives reflects protein oxidation. Data showed a significant increase in protein carbonylation (nearly 5.35 fold) upon 14 days of CHH exposure as compared to control group (p<0.001 vs. control) (Fig 2A).

Another known and proven marker of oxidative protein damage is AOPP in which ~9 fold increment was noted on 14 days CHH exposure (4.25±0.11 μmol Chloramine T mg/protein, p<0.001 vs. control) as compared to control group (0.47±0.03μmol Chloramine T mg/ protein) (Fig 2B). Accumulation of misfolded proteins enhanced the proteolytic activity of 20S proteasome, a catalytic core of 26S proteasome that was significant increase by 6.52 and 9.07 fold (p<0.01 vs. control) in chymotrypsin-like protease activity of 20S proteasome in 07 days and 14 days CHH exposed rats in relation to control rats respectively (Fig 2C).

Calpain, Ca^2+^ -activated proteases was also found to be increased by 5.54-fold on 14days CHH exposed animals with the comparison to control animals (p<0.001 vs. control) (Fig 2D).

Ca^2+^ ions are the regulatory factor in many signaling processes. The present study described intracellular calcium level as it regulated the calpain activity, nearly 2-fold increase was observed at 01 day and 03 day CHH exposure in comparison to control rats; while, a significant and highest amount of [Ca^2+^]_i_ (~ 2.7 and 3-fold) increase was monitored at 07 and 14days CHH exposure (Fig 2E) in relation to control rats.

Since cellular stress resulted in activation of ER chaperones to deal with misfolded and unfolded proteins, we examined changes in GRP-78 and PDI protein levels, involved in post-translational modification and known to be up-regulated with disrupted ER homeostasis. GRP-78 was upregulated nearly 2.2 fold at 07d CHH exposure (p<0.001) and increased further to 2.6 fold at 14d of CHH exposure in comparison to control group. Increased expression of PDI was also observed, nearly 1.4 fold but only at 14 days CHH exposure as compared to control (p<0.001) (Fig 2F–2H).

### Effect of chronic hypobaric hypoxia on protein synthesis signaling

**Fig 3:**
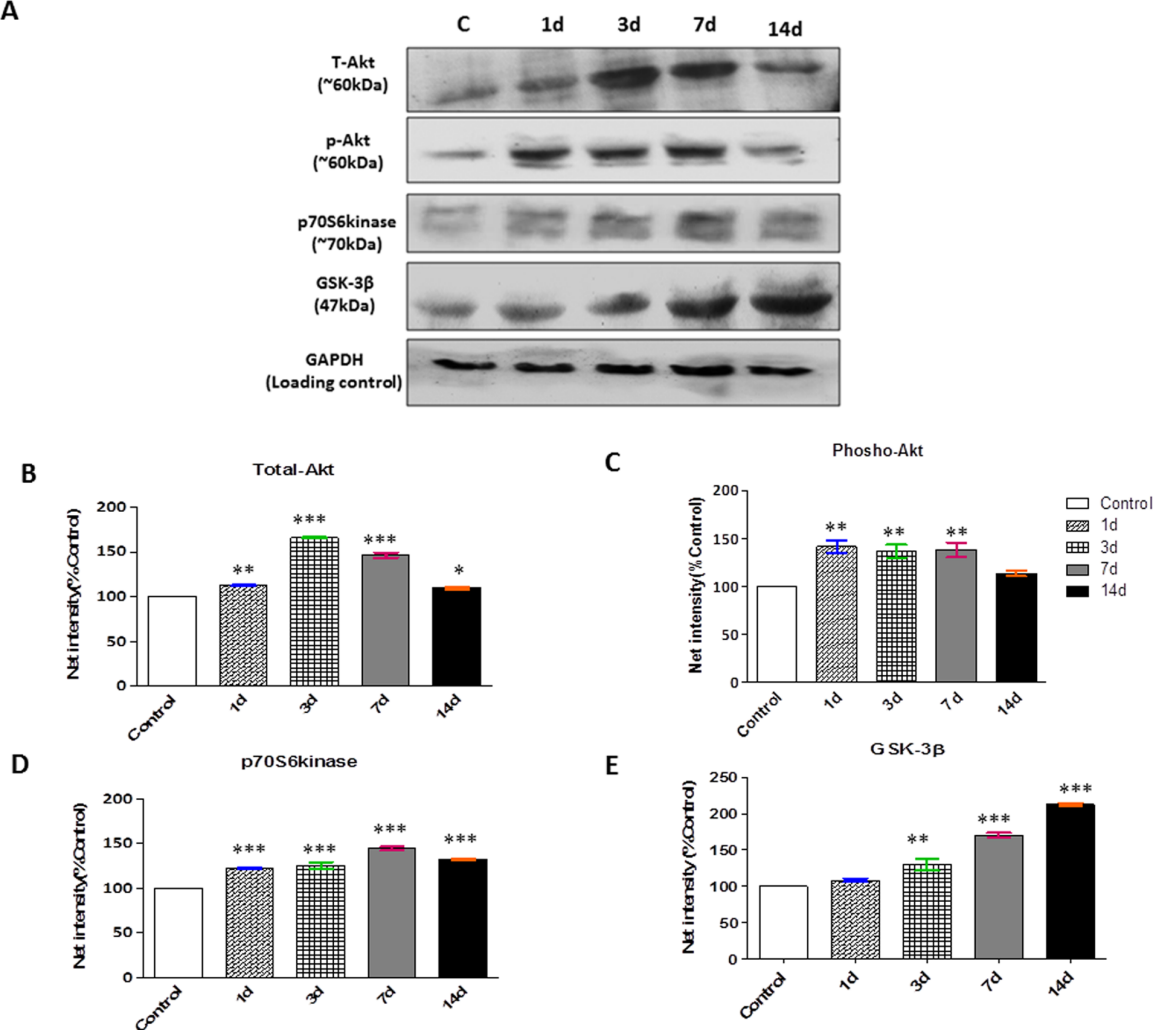
Anabolic signalling / protein translational machinery response under chronic hypoxia insult. **(A)** Representative western blots for expression of protein synthesis cascade, in skeletal muscles tissues cytoplasmic extracts. GAPDH considered as the loading control and (**B-E)** Semiquantitative analysis of the expression of total-Akt, p-Akt, p70S6kinase and GSK-3β respectively is presented in the graphs. The densitometric analysis is shown as mean with standard error (bars) performed in n = 3 independent experiments. *p<0.05 Vs. Control; **p<0.01 Vs. Control; ***p<0.001 Vs. Control.

Signaling molecules involved in protein translational machinery showed a marginal increase in a time-dependent manner of CHH exposure. Further, total-Akt, p-Akt (phosphorylated Akt), glycogen synthase kinase (GSK-3β) and p70S6K were quantified by western blot (Fig 3A). The results delineated that Akt level was significantly increased by ~66% in 3d HH exposure but fell down onwards 07d HH and 14d HH exposure by 46% and 10% respectively (Fig 3B). Expression of p-Akt was up-regulated till 07 days CHH exposure (Fig 3C). Downstream regulator of protein synthesis, p70S6kinase, was gradually increased by 44% up to 07 days exposure (Fig 3D). One of the downstream or negative regulators of Akt, GSK-3β was also significantly increased in CHH exposure (Fig 3E). IGF-1 is a polypeptide hormone participates in muscle development, repair, and regeneration program. In the present study, no significant changes in IGF-1 levels were observed between control and hypoxic group (Table 1).

**Table 1:** Effect of chronic hypobaric hypoxia on insulin growth factor-1.

Control Rats	1day hypoxia	3days hypoxia	7days hypoxia	14days hypoxia
5.37±0.05	5.39±0.03	5.20±0.06	5.37±0.04	5.45±0.09

Data represents the mean±SEM; N = 5.Unit = ng/mg protein

### Effect of chronic hypobaric hypoxia on inflammatory signaling

**Fig 4:**
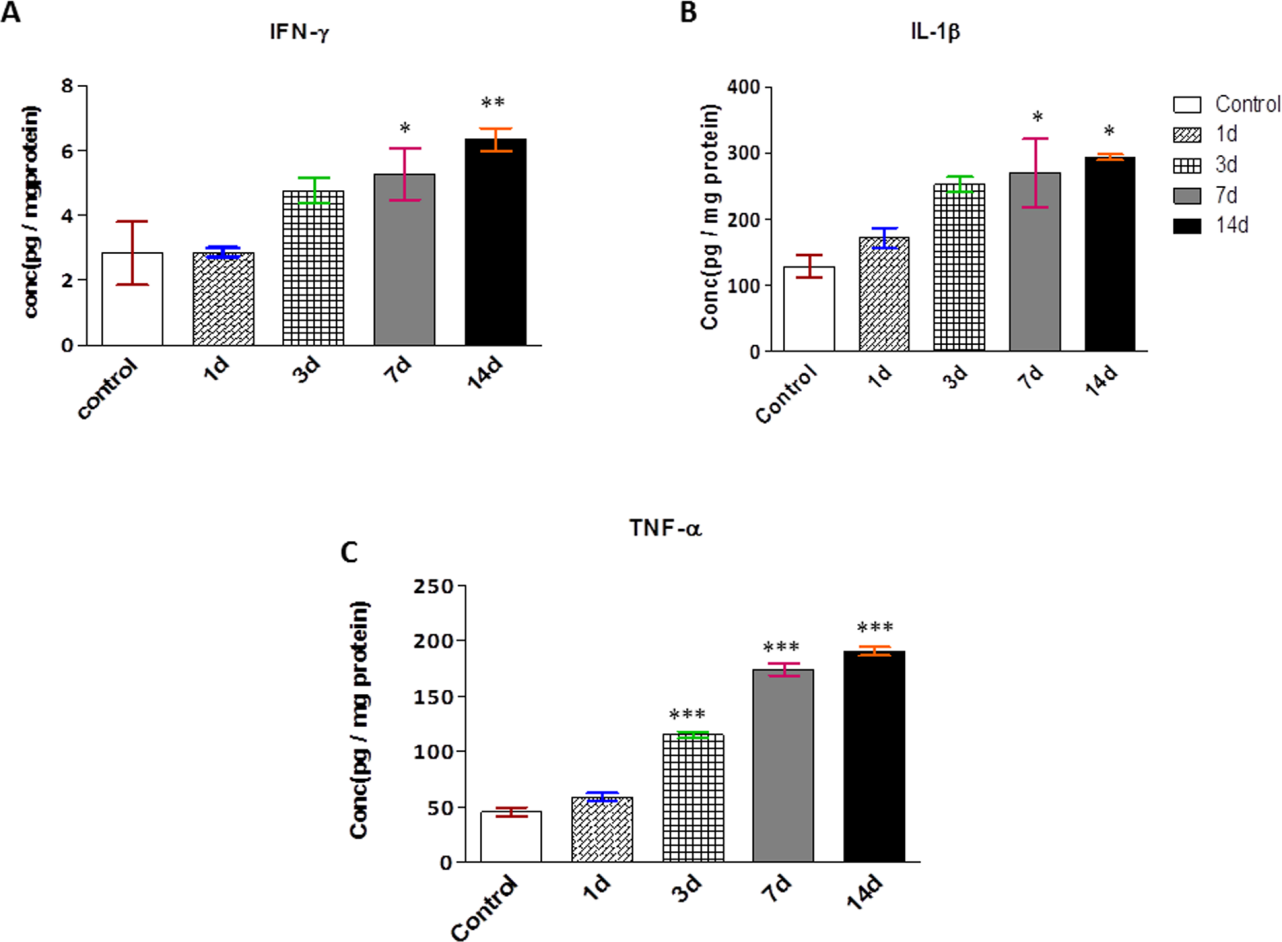
Effect of chronic hypoxia on inflammatory signaling. **(A)** IFN-ϒ **(B)** IL-1β, and **(C)** TNF-α. All values presented as mean ± SEM. *p<0.05 Vs. Control; **p<0.01 Vs. Control; ***p<0.001 Vs.Control.

Hypoxia leads to the generation of inflammation via activation of pro-inflammatory cytokines. The present study depicted the substantial elevation in IFN-γ, IL-1β, TNF-α levels on 14 days CHH exposure as compare to control rats (Fig 4A, 4B and 4C).

### Upregulated NF-κB and E3 ligase mediated protein degradation via proteasome pathway in response to chronic hypoxia

**Fig 5:**
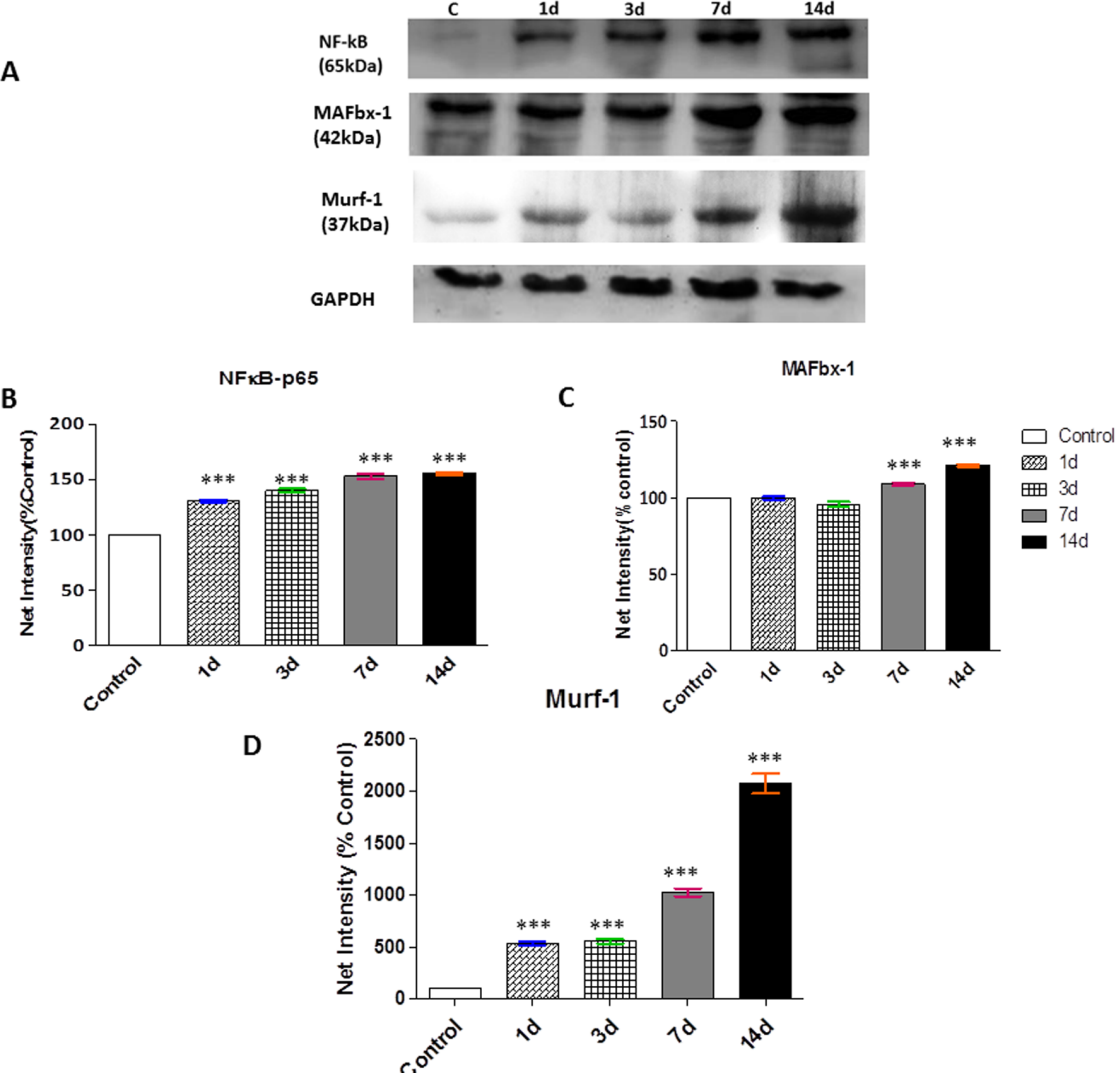
Upregulated NF-κB and E3 ligase mediated protein degradation in response to chronic hypoxia. **(A)** Representative western blots for expression of NF-κB and Mafbx-1 in skeletal muscles and **(B**—**D)** Semiquantitative analysis of the expression of NF-κB, MAFbx-1and MuRF-1 is presented in the graph. The densitometric analysis is shown as mean ± SEM performed in n = 3 independent experiments. *p<0.05 Vs. Control; **p<0.01 Vs. Control; ***p<0.001 Vs. Control.

In normal muscle physiological condition, NF-κB is localized in the cytoplasm forming a complex with its inhibitory protein IκB-a. Upon stressful condition, IκB-a cleaved from this complex and NF-κB translocated to the nucleus (NF-κB activation) where it enhanced gene transcription. The present study showed the upregulation in NF-κB expression in skeletal muscle in time-dependent CHH exposure. MAFbx-1 and MuRF-1 was also found to be increased in its expressions in a time-dependent manner of CHH exposure as compared to control group (Fig 5A–5D).

### Apoptotic signaling cascade

**Fig 6:**
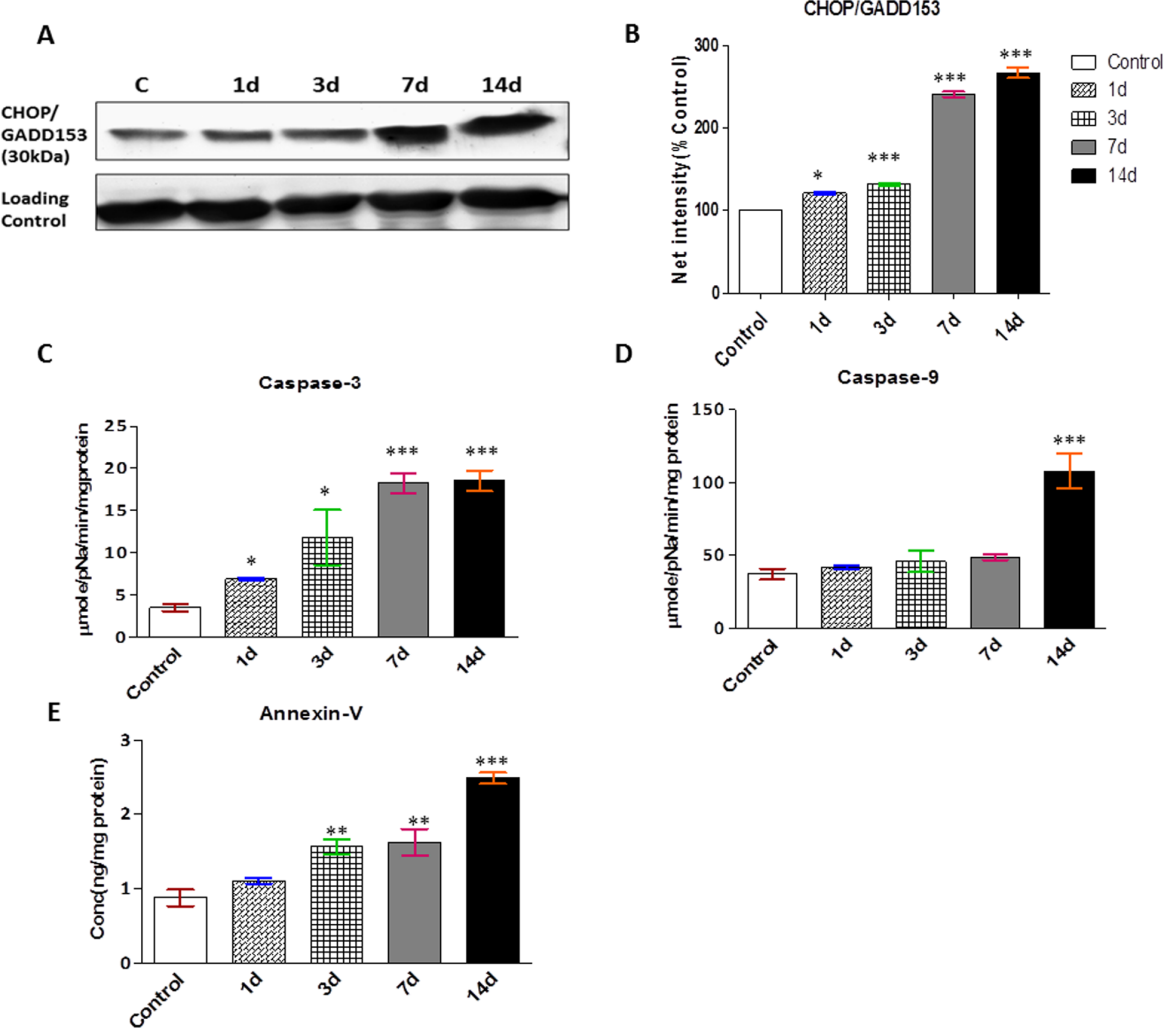
Chronic hypoxia led to the activation of apoptotic signaling cascade. **(A)** Representative western blots for expression of CHOP/GADD153 from the cytoplasmic extracts of skeletal muscle. **(B)** Semiquantitative analysis of the expression of ER associated degradation, CHOP/GADD153. GAPDH considered as the loading control. The densitometric analysis is shown as mean with standard error (bars) performed in n = 3 independent experiments. Specific colorimetric peptide substrate has been used for particular caspases in cellular fuction. Activity of caspase-3 and caspase-9 was measured using colorimetric substrate Ac-Asp-Glu-Val-Asp p-Nitroaniline and Ac-Leu-Glu-His-Asp-pNA. The cysteine protease activity of **(C)** Caspase-3 and **(D)** Caspase-9 cleaves the substrate and releases pNA, the absorbance of which can be measured at 405 nm, and the **(E)** Annexin-V was measured by ELISA kit. All values presented as mean ± SEM. *p<0.05 Vs. Control; **p<0.01 Vs. Control; ***p<0.001 Vs. Control.

CHH exposure also induced a marked upregulation in the expression of transcription factor C/EBP homologous protein (CHOP), associated with ER stress that initiated cell death in the skeletal muscle (Fig 6A and 6B). Apoptosis is one of the crucial processes involved in skeletal muscle atrophy. To explore the underlying mechanism of apoptosis, caspases and annexin V were estimated and the results showed a significant increase in caspase-3 and caspase-9 activity (~ 5.45 and 3 fold respectively) on 14d CHH exposure as compared to control rats (Fig 6C and 6D). Annexin-V, a potent marker of apoptosis was also found increased significantly from 3d to 14d in time-dependent CHH exposure (Fig 6E).

### Bioinformatics networking by STRING 10.0 Software

The STRING database was used to collect and integrate the information related to the cellular functional interaction between expressed proteins. The integration of protein-protein interaction includes direct (physical) interactions and indirect (functional) interactions as both are specific and biologically meaningful [24, 25, 26]. These associations derived from genomic context, co-expression, experiments and previous knowledge.

**Fig 7:**
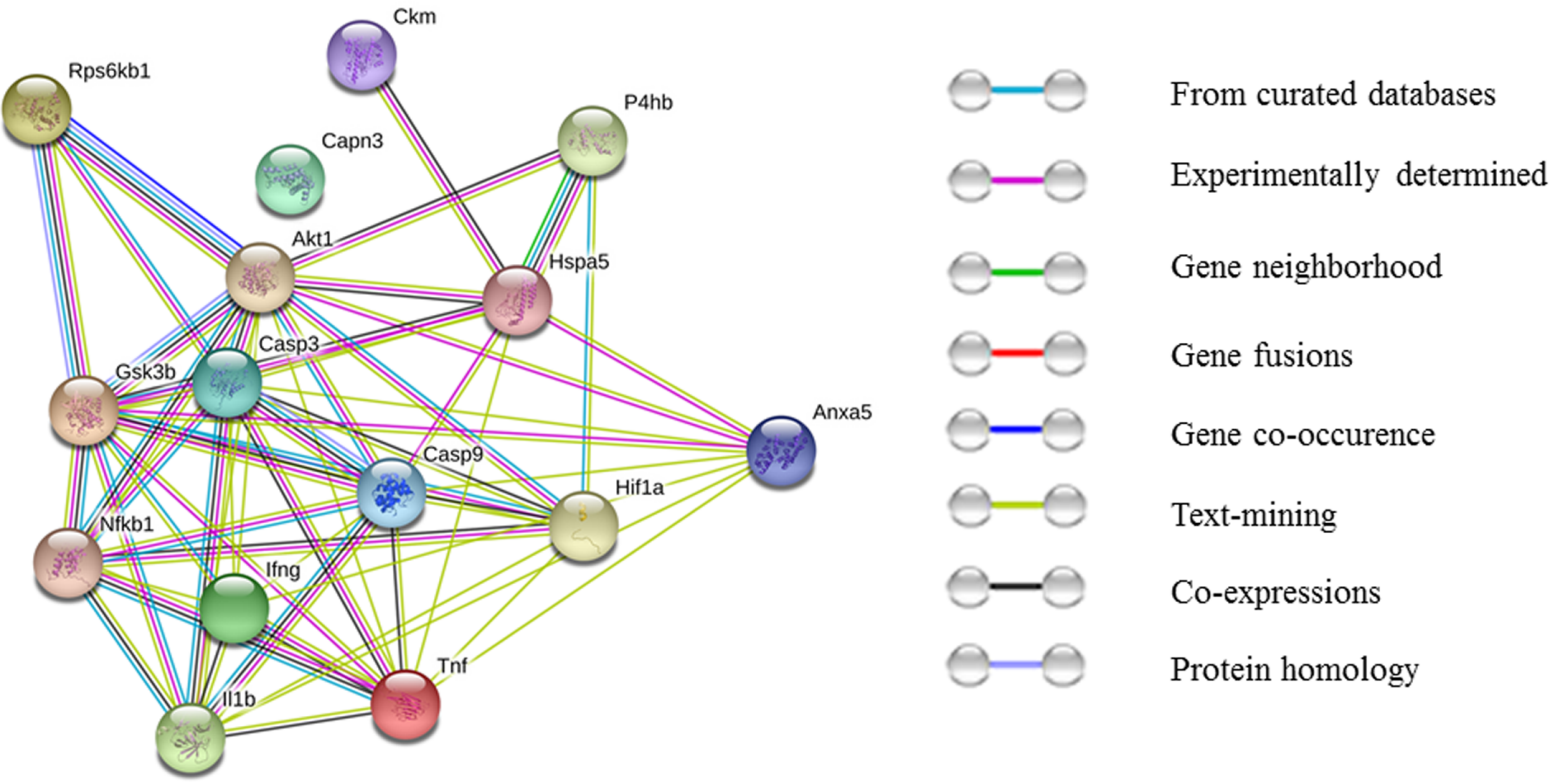
The network analysis of proteins differentiated expressed in chronic hypobaric hypoxia exposed groups using STRING 10.0.

In the present study, the proteins which showed statistically significant change were uploaded into the STRING 10.0 software to analyze the interactions of all the proteins (Fig 7). The score of confidence is considered for presenting between protein-protein interactions. According to STRING 10.0 software, 0.150 score for low, 0.400 score for medium, 0.700 score for high and 0.900 score for the highest confidence respectively. In the present study, we uploaded the proteins which significantly change during CHH exposure and among them 12 proteins had a high confidence and highest confidence interaction i.e. >0.700 confidence level. The score among these proteins is presented in Table 2. As per the score result, Tnf, NfκB1, Il1b, Ifng, Gsk3b, Casp9, Casp3, Akt1, Rps6κB1, Igf1, Hif1a, and Anxa5 showed high and highest confidence, which demonstrates that the above proteins have a close relationship and involved in CHH induced muscle protein loss.

**Table 2:** The score among the proteins. High confidence: 0.700; highest confidence: 0.900.

Node1	Node2	Node1 accession	Node2 accession	Score
Anxa5	Akt1	ENSRNOP00000019552	ENSRNOP00000038369	0.787
Anxa5	Casp3	ENSRNOP00000019552	ENSRNOP00000014096	0.911
Anxa5	Casp9	ENSRNOP00000019552	ENSRNOP00000017972	0.856
Casp3	Akt1	ENSRNOP00000014096	ENSRNOP00000038369	0.952
Casp3	Casp9	ENSRNOP00000014096	ENSRNOP00000017972	0.964
Casp3	Tnf	ENSRNOP00000014096	ENSRNOP00000001110	0.879
Casp9	Akt1	ENSRNOP00000017972	ENSRNOP00000038369	0.992
Casp9	Il1b	ENSRNOP00000017972	ENSRNOP00000006308	0.701
Casp9	Tnf	ENSRNOP00000017972	ENSRNOP00000001110	0.785
Gsk3b	Akt1	ENSRNOP00000003867	ENSRNOP00000038369	0.988
Gsk3b	Ifng	ENSRNOP00000003867	ENSRNOP00000009917	0.799
Gsk3b	Il1b	ENSRNOP00000003867	ENSRNOP00000006308	0.812
Gsk3b	Tnf	ENSRNOP00000003867	ENSRNOP00000001110	0.792
Hif1a	Akt1	ENSRNOP00000042230	ENSRNOP00000038369	0.782
Hspa5	Akt1	ENSRNOP00000025064	ENSRNOP00000038369	0.795
Hspa5	P4hb	ENSRNOP00000025064	ENSRNOP00000051841	0.939
Ifng	Il1b	ENSRNOP00000009917	ENSRNOP00000006308	0.884
Ifng	Tnf	ENSRNOP00000009917	ENSRNOP00000001110	0.943
Il1b	Casp3	ENSRNOP00000006308	ENSRNOP00000014096	0.775
Il1b	Nfkb1	ENSRNOP00000006308	ENSRNOP00000028944	0.976
Il1b	Tnf	ENSRNOP00000006308	ENSRNOP00000001110	0.969
Nfkb1	Akt1	ENSRNOP00000028944	ENSRNOP00000038369	0.898
Nfkb1	Tnf	ENSRNOP00000028944	ENSRNOP00000001110	0.961
Rps6kb1	Akt1	ENSRNOP00000005226	ENSRNOP00000038369	0.944

## Discussion

High altitude hypoxia imposes several physiological, metabolic and biochemical changes disturbing skeletal muscle homeostasis. A shift in the redox potential under hypobaric hypoxia leads to activation or impairment of key biological cascades, regulated intricately by proteins. Proteostasis is a complex interacting system which consist of diverse biological pathways including protein synthesis, folding, trafficking, disaggregation, and degradation [27]. Prolonged and diverse environmental stress represent a constant threat to normal protein folding in the cells. Even though the presence of oxidative protein modifications under high altitude induced stressors is known, few studies have scrutinized the disturbance of proteostasis in the context of high altitude milieu [28, 29].

In the present study, our results showed a significant decline in wet muscle weight and tibial length ratio by 23% on 14d CHH which evidently indicated skeletal muscle atrophy in response to severe and prolonged hypobaric hypoxia. Further, skeletal muscle creatine phosphokinase activity (CPK), the main energy reservoir, was found decreased on 14d CHH exposure. This result clearly indicate the membrane injury surrounding muscle cells and causing leakage of CPK into the bloodstream on CHH exposure. The literature stated reversible modifications like glutathionylation, nitrosylation, and carbonylation are covalent oxidative modification contributed to the misfolding and damage of protein structure [30, 31, 32]. Evidence of oxidative protein damage on high altitude exposure has since long been corroborated in human as well as animals, indicating the susceptibility of cellular proteins to hypobaric hypoxia [33, 34]. Carbonyl formation is considered as an early marker for oxidative protein damage. The most likely amino acid residues to form carbonyl derivatives are lysine, arginine, proline and threonine. In our study, we found an enhance level of protein carbonylation and AOPP by ∼5.35 and ∼9 fold respectively over 14d CHH exposure. Overload oxidized/misfolded proteins triggered the activation of molecular chaperones for maintaining cellular homeostasis. Having established an enhanced presence of oxidized proteins, we identified the expression of the major ER chaperones, GRP-78 and PDI which was also upregulated in a time-dependent manner of CHH exposure. These results suggested that although chaperones were activated but the oxidative damage to proteins was too severe which ultimately led to the activation of several degradative pathways in response to CHH exposure.

Dudek and co-workers [35] reported that carbonyl-containing oxidized proteins were selectively removed by ubiquitinating machinery, UPS. Although skeletal muscle atrophy probably not regulated by a single mechanism, but a complex one, the calcium-dependent cysteine proteases, calpain also considered as one of the main culprit of protein degradation [36]. A growing evidence depicted that both calpain and the ubiquitin-proteasome system act synergistically and in a coordinated manner during sepsis induced muscle proteolysis [37]. The present study findings also described a significant increase in calpain activity on 14d CHH exposure, which was accompanied by an increase in intracellular [Ca^2+^] (~3 fold), which is rate limiting co-factor of calpain. These results also placed an harmony with the previous reports related to other muscular pathologies such as dystrophy, sepsis and denervated muscles [38, 39].

Muscle atrophy, a consequence of net protein loss due to the imbalance of protein metabolism towards increase protein breakdown [2, 3]. IGF1/Akt pathway is unique as it controls both protein synthesis and protein degradation. Léger et al. [39] reported the role of Akt, mTOR and GSK-3β in the regulation of skeletal muscle mass. GSK-3β is the negative regulator of Akt, participates in causing muscle atrophy. Recent study by Chaillou et al. [40] have reported that impaired muscle regeneration during hypobaric hypoxia (5,500m) was associated with a blunted activation of p70S6K, mTOR and 4E-BP1 phosphorylation on 03d of regeneration and higher activation of p70S6kinase at 07d. Furthermore, they reported the marked increase in the mRNA levels of E3ligases after 07d of hypoxia which clearly showed reduction in protein synthesis and increased myofibrillar protein break down. Results of the present study are in agreement with these findings by showing the significant increase in key regulator of protein synthesis, Akt, p-Akt, p70S6kinase during first 07d of hypobaric hypoxia. In addition to these findings, our study reported that GSK-3β, downstream molecule in IGF-I/Akt signaling was also upregulated in 03d of CHH exposure which remain activated till 14d, indicating its role in protein degradation. While, Chaillou et al. [40] studied the effect of ambient hypoxia which enhanced the loss of muscle mass after extensive injury on the other hand our study depicted the probable signalling mechanisms for hypobaric hypoxia induced muscle protein loss in healthy adult rats.

A progressive loss of skeletal muscle mass and strength, coupled with oxidative stress and inflammation associated with skeletal muscle diseases like disuse, aging, cancer cachexia and denervation [41]. Recent reports also detailed the vital role of inflammation in altitude-related illness. Plasma TNF-α, IL-1β and IL-6 levels significantly increased when volunteers ascended to an altitude of 3860 m [42]. To the best of our knowledge, first time our study reported the increase pro-inflammatory cytokines (TNF-α, IFN-© and IL-1β) in skeletal muscles in response to chronic hypoxic insult.

Additionally, enhanced TNF-α also activated NF-κB in hypoxic skeletal muscle. Beside this, muscle specific E3 ligases, Mafbx-1 and MuRF-1 was also significantly increased on 07d which remain elevated till 14d of CHH which directly reflects the regulation of proteasome pathway by atrogenes. The findings of our study clearly indicated that severe hypoxia augments inflammation, a key determinant in the progression of muscle atrophy.

The activation of ER chaperones provided protection to the cell, but severe stress or misfolding triggered ER-associated cell death. The contribution of cell death has now been reported in various diseases, including cancer, cachexia, ischemic stroke, and multiple sclerosis [43, 44, 45]. The present study also depicted the progressive upregulation of CHOP, which became significant in a time-dependent manner of CHH exposure. Activated UPS and calpain pathways account for removal of misfolded/oxidized proteins and activation of apoptotic signalling cascade in the cell. The present study observed an increase in caspase-3, caspase-9 and annexin V activity which suggested increase muscle protein breakdown 14d CHH exposure and these results are in accordance with the previous reports [46, 47]. These observations represent another paradigm which envisages the activation of all three proteolytic systems i.e. UPS, calpain, and caspase-3 in response of prolonged and severe environmental hypoxic condition.

Further, string analysis predicted protein-protein interactions among the proteins which were also analysed via western or biochemical in CHH exposed rats vs control rats. To best of our knowledge, this is the first time the data reported CHH induced muscle atrophy via string pathway which were further proved by *in-vivo* analysis. Based on the study, 15 proteins was depicted a high confidence and highest confidence interaction i.e. >0.700 confidence level. As per the score result, Tnf, P4hb, NfκB1, Il1b, Ifng, Hspa5, Gsk3b, Casp9, Casp3, Anxa5, Akt1, Rps6kb1, Ckm, Hif1a and Capn3 showed high and highest confidence and the details of each gene also presented (as shown in Table 2 and Table 3). The genes which were showing high and highest confidence interaction played an important role in skeletal muscle atrophy. Gsk3b, Akt1 and Rps6kb1 related to protein synthesis pathway, Hif1a considered as the master transcriptional regulator of cellular and developmental response to hypoxia, P4hb is a multifunctional protein involved in catalysis of the formation, breakage and rearrangement of disulfide bond, Ckm known for muscle damage and hspa5 related to GRP78 protein engaged with ER response. These genes showed a high confidence value hence it is predicted that cell survival pathway triggered during chronic hypobaric hypoxic exposure. Further, Tnf, Il1b and Ifng related to inflammatory response, NfκB1 controls transcription of DNA, cytokine production and cell survival, Casp9, Casp3 and Anxa5 correspond to apoptosis process. The above said genes showed a highest confidence interaction via string analysis and the results were further proved via *in-vivo* experiments and interestingly both the results made concurrence with each other. Ergo, the analogous results could provide a clear indication that inflammation and apoptosis are the main responsible pathways that lead to CHH induced skeletal muscle protein loss.

**Table 3:** The details of gene.

Gene	Protein
Tnf	Tumor necrosis factor
Rps6kb1	Ribosomal protein S6 kinase beta-1
P4hb	Protein disulfide-isomerase
Nfkb1	Nuclear factor NF-kappa-B
Il1b	Interleukin-1 beta
Igf1	Insulin-like growth factor 1
Ifng	Interferon gamma
Hspa5	78 kDa glucose-regulated protein
Gsk3b	Glycogen synthase kinase-3 beta
Casp9	Caspase-9
Casp3	Caspase-3
Anxa5	Annexin A5
Akt1	serine/threonine- protein kinases
Ckm	Creatine kinase M-type
Capn3	Calpain-3

**Fig 8:**
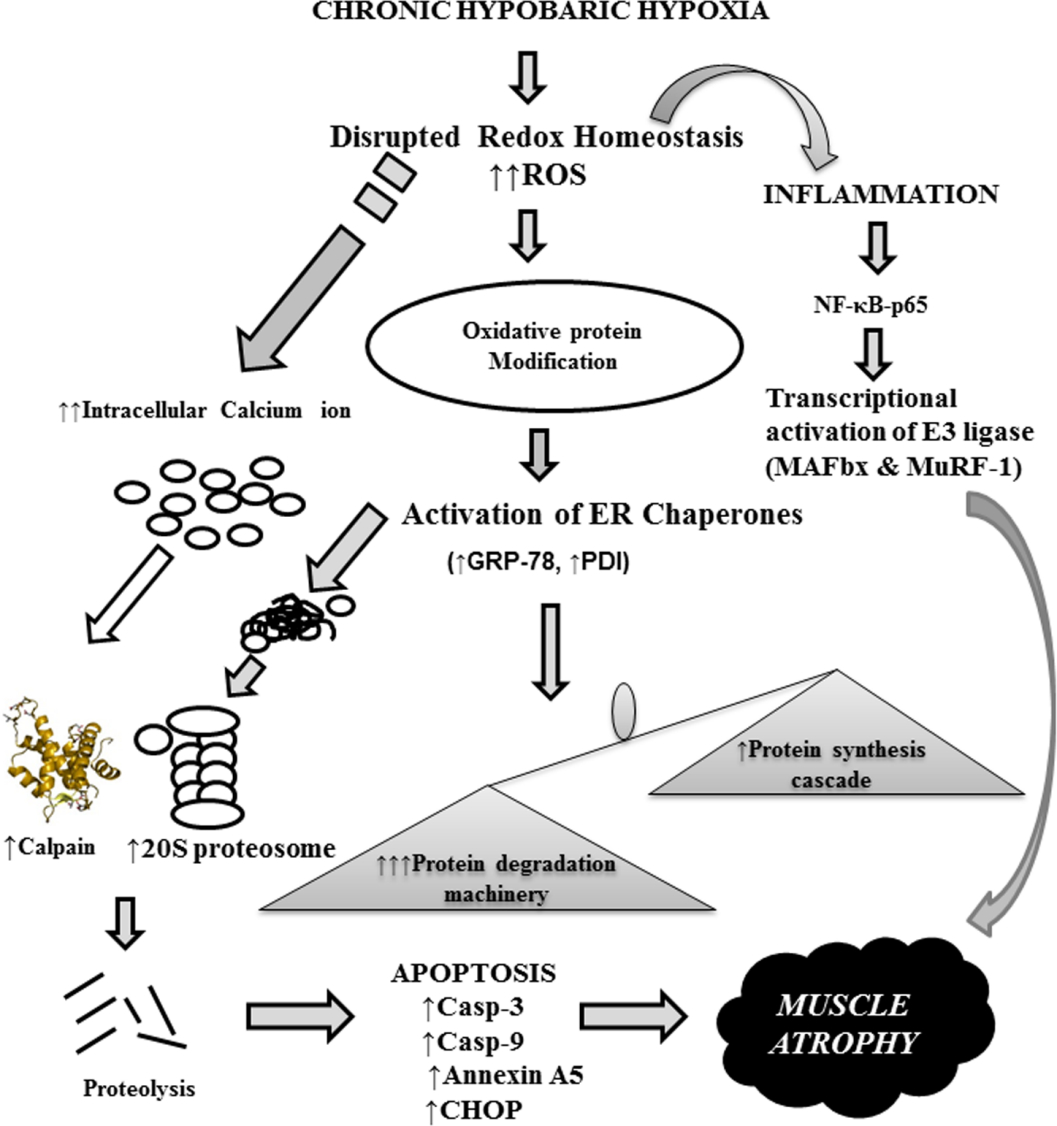
Diagrammatic representation of mechanism approach to elucidate the chronic hypobaric hypoxia induced muscle atrophy.

Conclusively, our findings provide molecular insight into the development of progressive muscular atrophy due to the perturbed protein quality control machinery under prolonged and severe hypoxia. The enhanced skeletal muscle protein loss responsible due to proteome imbalance, free radical mediated inflammation and impaired protein synthesis flux. Fig 8 clearly demonstrated that hypobaric hypoxia induced skeletal muscle atrophy is a multifaceted pathophysiological condition which was regulated by multiple signaling pathways. The leads from the current study might help in the development of proteostasis modulators as therapeutic interventions which could restore proteostasis in multiple muscle related pathologies and may act against all proteolytic systems involved during atrophy.

## Supporting information

S1 Table Details of primary antibodies. (DOC)

S2 Table Details of secondary antibodies. (DOCX)

S3 Table Fluorimetric/Colorimetric substrate used for enzyme activity assay. (DOCX)
