# Supplementary material for: Role of altered proteostasis network in chronic hypobaric hypoxia induced skeletal muscle atrophy
Source: PLoS One. 2018 Sep 21;13(9):e0204283. doi: 10.1371/journal.pone.0204283 (PMC6150520; doi:10.1371/journal.pone.0204283)
Supplement: S1 Table — (DOC) [file pone.0204283.s001.doc]

**S1 Table. Details of** Primary Antibodies

| S.No. | **Antibody** | **Description** | **Source** | **Manufacturer** | **WB Dilution** |
| --- | --- | --- | --- | --- | --- |
| 1. | GRP-78 | Polyclonal  (rabbit) | G9043 | Sigma  (St. Louis, MO, USA) | 1:3000 |
| 2. | PDI | Monoclonal  (Mouse) | Sc-74551 | Santa Cruz Biotech | 1:800 |
| 3. | t-Akt(1/2/3) | Polyclonal  (rabbit) | Sc-8312 | Santa Cruz Biotech | 1:1000 |
| 4. | p-Akt | Polyclonal  (rabbit) | Sc-7985 | Santa Cruz Biotech | 1:800 |
| 5. | p70S6kinase | Polyclonal (rabbit) | 9202S | Cell signalling Technology | 1:1000 |
| 6. | GSK-3β | Polyclonal (rabbit) | G7914 | Sigma  (St. Louis, MO, USA) | 1:2000 |
| 7. | NF-kBp-65 | Monoclonal  (Mouse) | N8523 | Sigma  (St. Louis, MO, USA) | 1:1000 |
| 8. | Fbx32 | Monoclonal  (Rabbit) | ab168372 | Abcam’s Rab Mab Technology | 1:1500 |
| 9. | MuRF-1 | Polyclonal  (Rabbit) | ab183094 | Abcam’s Rab Mab Technology | 1:1500 |
| 9. | CHOP/GADD153 | Polyclonal (rabbit) | Sc-575 | Santa Cruz Biotech | 1:1000 |
| 10. | GAPDH  (Loading Control) | Monoclonal  (mouse) | Sigma-G8795 | Sigma  (St. Louis, MO, USA) | 1:6000 |

WB = Western blotting
