# Supplementary material for: Role of altered proteostasis network in chronic hypobaric hypoxia induced skeletal muscle atrophy
Source: PLoS One. 2018 Sep 21;13(9):e0204283. doi: 10.1371/journal.pone.0204283 (PMC6150520; doi:10.1371/journal.pone.0204283)
Supplement: S2 Table — (DOCX) [file pone.0204283.s002.docx]

**S2 Table. Details of Secondary Antibodies**

| **S.No.** | **Antibody** | **Description** | **Source** | **Manufacturer** | **WB Dilution** |
| --- | --- | --- | --- | --- | --- |
| 1. | Anti-mouse | Polyclonal (rabbit) | Sigma-A9044 | Sigma  (St. Louis, MO, USA) | 1:25000 |
| 2. | Anti-rabbit | Polyclonal (goat) | Sigma-A9169 | Sigma  (St. Louis, MO, USA) | 1:80000 |

WB= Western blotting
