# Supplementary material for: Role of altered proteostasis network in chronic hypobaric hypoxia induced skeletal muscle atrophy
Source: PLoS One. 2018 Sep 21;13(9):e0204283. doi: 10.1371/journal.pone.0204283 (PMC6150520; doi:10.1371/journal.pone.0204283)
Supplement: S3 Table — (DOCX) [file pone.0204283.s003.docx]

**S3 Table. Fluorimetric/ Colorimetric Substrate used for enzyme activity assay**

| **S.No.** | **Protein/Enzymes** | **Substrate** | **Source** | **Type** |
| --- | --- | --- | --- | --- |
| **1.** | 20S proteasome | Succ-LLVY | Sigma S4939 | Fluorimetric |
| **2.** | Calpain | SLY-AMC | Sigma S1153 | Fluorimetric |
| **3.** | Caspase-3 | Substrate-I | Calbiochem 235400 | Colorimetric |
| **4.** | Caspase-9 | Substrate-II  (Ac-LEHD-pNA) | Calbiochem  218805 | Colorimetric |
